# Rapidly diverging evolution of an atypical alkaline phosphatase (PhoA^aty^) in marine phytoplankton: insights from dinoflagellate alkaline phosphatases

**DOI:** 10.3389/fmicb.2015.00868

**Published:** 2015-08-25

**Authors:** Xin Lin, Lu Wang, Xinguo Shi, Senjie Lin

**Affiliations:** ^1^State Key Laboratory of Marine Environmental Science, Xiamen UniversityXiamen, China; ^2^Department of Marine Sciences, University of ConnecticutGroton, CT, USA

**Keywords:** dinoflagellate, alkaline phosphatase, gene duplication, evolution, ecological strategy

## Abstract

Alkaline phosphatase (AP) is a key enzyme that enables marine phytoplankton to scavenge phosphorus (P) from dissolved organic phosphorus (DOP) when inorganic phosphate is scarce in the ocean. Yet how the AP gene has evolved in phytoplankton, particularly dinoflagellates, is poorly understood. We sequenced full-length AP genes and corresponding complementary DNA (cDNA) from 15 strains (10 species), representing four classes of the core dinoflagellate lineage, Gymnodiniales, Prorocentrales, Suessiales, and Gonyaulacales. Dinoflagellate AP gene sequences exhibited high variability, containing variable introns, pseudogenes, single nucleotide polymorphisms and consequent variations in amino acid sequence, indicative of gene duplication events and consistent with the “birth-and-death” model of gene evolution. Further sequence comparison showed that dinoflagellate APs likely belong to an atypical type AP (PhoA^aty^), which shares conserved motifs with counterparts in marine bacteria, cyanobacteria, green algae, haptophytes, and stramenopiles. Phylogenetic analysis suggested that PhoA^aty^ probably originated from an ancestral gene in bacteria and evolved divergently in marine phytoplankton. Because variations in AP amino acid sequences may lead to differential subcellular localization and potentially different metal ion requirements, the multiple types of APs in algae may have resulted from selection for diversifying strategies to utilize DOP in the P variable marine environment.

## Introduction

Alkaline phosphatase (AP) is a hydrolase [EC 3.1.3.1] that releases inorganic phosphate (P*i*) from various types of phosphoester molecules. In the marine ecosystem, AP plays a critical role as it enables bacteria and phytoplankton to meet the P-nutrient requirement from dissolved organic phosphorus (DOP) when the energetically preferred dissolved inorganic phosphate (DIP) is scarce. As such, AP activity has widely been adopted as an indicator of DIP limitation (Kolowith et al., 2001; Dyhrman et al., 2007). Many studies have been carried out to identify and characterize the AP gene in marine microorganisms, leading to the categorization of three types of APs in marine prokaryotes, but APs in marine eukaryotic phytoplankton have remained largely uncategorized.

The three types of APs documented in prokaryotes, PhoA^EC^, PhoX, and PhoD, share weak sequence homology with each other and exhibit different metal ion requirements for their active sites, different subcellular localizations and various substrate preferences (Luo et al., 2009; Sebastián and Ammerman, 2009; White, 2009; Kathuria and Martiny, 2011). Typical AP, usually referred to as PhoA^EC^, was among the first isolated from *E. coli* and characterized as homodimeric phosphomonoesterase with phosphomonoesters as the major substrate but also showing lower activity on phosphate diesters (Zalatan et al., 2006). Yet many other enzymes (e.g., phosphodiesterase and phosphoglycerate mutases) with a similar mechanistic function as PhoA^EC^ exist in a wide range of organisms. Because their folded protein structures share a conserved bimetal binding core (2Zn-Mg), PhoA^EC^ and these other metalloenzymes are grouped into an AP superfamily, which has been adopted as a model to study the relationship between evolutionary driving forces and divergent enzymatic functions (Galperin and Jedrzejas, 2001; Zalatan et al., 2006). The promiscuity of PhoA^EC^ for substrates (both monoester and diester) is suggested to have resulted from selective advantages in evolutionary optimization through gene duplication of an ancestral phosphohydrolase gene (Zalatan et al., 2006).

PhoD and PhoX are also able to hydrolyze both monoesters and diesters, and have been reported to be dependent on Ca and Ca-Fe, respectively (Zaheer et al., 2009; Kageyama et al., 2011; Sebastian and Ammerman, 2011; Yong et al., 2014). Contrary to the limited reports of PhoA^EC^ amongst marine microbial communities (Luo et al., 2009, 2010), the Ca-dependent PhoX and PhoD appear to be much more common, presumably an adaption to Zn limitation in the ocean (Sebastian and Ammerman, 2011). Comparative genomic data reveals five different families of phosphatases in different cyanobacterial strains, amongst which there is an atypical PhoA-like AP, PhoA^aty^, so named because it is distantly related to PhoA^EC^ (Ray et al., 1991; Moore et al., 2005; Orchard et al., 2009; Scanlan et al., 2009).

Growing genomic data provides evidence for the presence of different types of AP genes in eukaryotic phytoplankton (Armbrust et al., 2004; Dyhrman et al., 2012; Lin et al., 2012a, 2013), though only some of these have ever been (partially) characterized through gene isolation and expression. Putative *phoX* genes have been identified in the green algae, *Volvox carteri* and *Chlamydomonas reinhardtii* (Quisel et al., 1996; Hallmann, 1999; Moseley et al., 2006). Moreover, there are several novel (uncategorized) types of APs, e.g., EHAP1 of the haptophyte *Emiliania huxleyi* (Xu et al., 2006), an apparently Ca-dependent putative AP from the pelagophyte *Aureoumbra lagunensis* (Sun et al., 2012) and the diatom *Phaeodactylum tricornutum* (Bowler et al., 2008; Lin et al., 2013), and AP genes isolated from dinoflagellates (Lin et al., 2011, 2012a,b; Morey et al., 2011). Because of their highly divergent sequences, there is an increasing need for structural and functional classification of these APs. Besides, the evolutionary mechanism underlying the high variability of these diverse APs in marine phytoplankton needs to be characterized.

In this study, we attempted to classify APs in eukaryotic phytoplankton and gain an understanding of how the AP gene in this group of organisms has evolved, using dinoflagellates as a case study. We analyzed AP genes from 15 strains, representing four classes of the core dinoflagellates, Gymnodiniales Prorocentrales, Suessilaes, and Gonyaulacales. Existing AP sequences from both prokaryotic and eukaryotic phytoplankton were also collected from various public databases for phylogenetic analysis and sequence comparison. Our analyses indicated that dinoflagellate APs were only similar to PhoA^aty^-like APs reported in cyanobacteria, and that they have undergone gene duplication within species. We also discuss the evolutionary relationships amongst the multiple types of APs found in marine phytoplankton.

## Materials and methods

### Algal culture

Fifteen dinoflagellate strains used in this study (Table 1) were provided by the Center for Collections of Marine Bacteria and Phytoplankton, Xiamen University (CCMBP), Provasoli-Guillard National Center for Marine Algae and Microbiota (NCMA), and Jinan University. These strains were cultured in sterilized oceanic seawater (filtered through 0.22 μm pore size filters, 30 psu) enriched with the full nutrient regime of the L1 or f/2 medium (Guillard and Ryther, 1962; Guillard, 1975; Guillard and Hargraves, 1993) or the same nutrient regime except for the reduced phosphate concentration (2 μM), in a temperature controlled incubator at 15 or 20°C and under a 12:12 or 14:10 light dark cycle with a photon flux of 100 μE m^−2^ S^−1^. The identity of each strain was confirmed by amplifying the ITS ribosomal intergenic spacer region using 18ScomF-3end and com28SR1 primer pair (Wang et al., 2014).

**Table 1:** **Summary of the dinoflagellate AP characteristics**.

**Class**	**Species**	**Gene (Protein)**	**ORF (bp)**	**Amino acid**	**Signal peptide**	**GPI anchor**	**Intron**	**Protein variants**	**Subcellular localization**
Gymnodiniales	*Amphidinium carterae* CCMP1314^a^	*acaap* (ACAAP)	2115	704	+	+	−	1	Extracellular
	*Karenia brevis* CCMP2229^a^	*kbrap* (KBRAP)	2061	686	+	+	−	6	Extracellular
	*Karenia mikimotoi* C32-HK	*kmiap* (KMIAP)	2043	680	+	+	+	1	Cytoplasmic
Prorocentrales	*Prorocentrum minimum* (Pavilard) Schiller 1933 CCMA15	*pmnap* (PMNAP)	2094	697	+	+	+	3	Extracellular
	*Prorocentrum donghaiense*^b^ CCMA190	*pdoap* (PDOAP)	2127	709	+	+	/	/	Extracellular
	*Prorocentrum micans* CCMA 143^c^	*pmicap* (PMICAP)	1463	487	/	/	/	5	/
Suessiales	*Symbiodinium* sp. Clade E CCMA192	*symEap*	2004	667	+	+	−	1	Extracellular
	*Symbiodinium kawagutii* CCMP2468^b^ Clade F	*skaap* (SKAAP)	1725	574	−	−	/	/	Extracellular
Gonyaulacales	*Alexandrium catenella* (Clade IIC) ACHK-NT^a^	*alecaap* (ALECAAP)	2181	726	+	+	−	1	Extracellular
	*Alexandrium catenella* (Clade IIC) ACHK-T^c^						/	1	Extracellular
	*Alexandrium catenella* (Clade IIC) ATDH02^c^						/	1	Extracellular
	*Alexandrium catenella* (Clade IIC) ATMJ01^c^						/	1	Extracellular
	*Alexandrium catenella* (Clade IIC) ATCI01^c^	*ATCI01-ap*	2181/2193	726/730	+	+	/	6	Extracellular
	*Alexandrium tamarense* (Clade IIB) CCAP1119/1	*ataap* (ATAAP)	2055/2160/2172/2184	684/719/723/727	+	+	−	36	Extracellular, Cytoplasmic, Chloroplast,
	*Alexandrium fundyense* (Clade I) CCMP1719	*afuap* (AlefuAP)	2124/2136/2142/2166	707/711/713/721	+	+	−	11	Plasma membrane

*“/,” Not examined; “+,” Presence; “−,” Absent*.

a *Data acquired from previous reports (Lin et al., 2011, 2012a,b)*.

b *Unpublished transcriptome data*.

c *ORF predicted based on gDNA*.

### Nucleic acid isolation and cDNA synthesis

Cells were collected from cultures during both the nutrient-replete exponential phase (genomic DNA extraction) and the phosphorus-depleted stationary phase (total RNA extraction) by centrifugation at 4000 × g for 15 min at 4°C. Cell pellets were re-suspended in DNA lysis buffer (10 mM Tris pH 8.0; 100 mM EDTA pH 8.0; 0.5% (w/v) SDS; 100 μg/ml proteinase K) for genomic DNA (gDNA) isolation and Tri-Reagent for total RNA isolation. Extraction of genomic DNA and total RNA was performed as previously reported (Zhang and Lin, 2005). The 1st-strand cDNA was synthesized from 300 ng of the total RNA using M-MLV reverse transcriptase (Promega, Madison, WI, USA) with a modified 454BT7-dT primer (Supplementary Table 1).

### Amplification of the full-length alkaline phosphatase cDNA and genomic DNA (gDNA) sequences

Degenerate primers DinoAPNF4a/b and DinoAPNR6/7a/7b (Supplementary Table 1) were used to obtain a fragment of the AP gene from either the gDNA or cDNA of different dinoflagellate species following previously published PCR conditions (Lin et al., 2012a). Amplified fragments were cloned into a T-vector and sequenced using the Sanger method (at least 10 clones for each sample). Once this partial sequence was annotated as an alkaline phosphatase gene using BLASTX against the GenBank non-redundant protein database, gene specific primers were designed to amplify both the 5′ and 3′ cDNA ends using the rapid amplification of cDNA ends (RACE) technique. In general, the reverse primers were paired with DinoSL (a unique 22-nt spliced leader only exists at the 5′ end of the mature mRNA transcript in dinoflagellate) to perform the 5′ RACE, and the forward primers were paired with 454BT7 (the adaptor attached after dT used for cDNA synthesis) to perform the 3′ RACE (Zhang et al., 2007; Lin et al., 2011). Then, the specific primers (Supplementary Table 1) were designed based on the sequence of 5′ UTR and 3′ UTR regions to amplify full-length ORFs of each AP gene from cDNA and gDNA. All the sequences acquired in this study have been deposited in GenBank under the accession numbers KT274032-KT274169.

### Analyses of introns, single nucleotide polymorphisms, and deduced protein sequences

Introns were detected by comparing the AP coding gene sequence of gDNA with the corresponding cDNA. Exon-intron boundaries were recorded in each case. In analyzing sequence polymorphisms, we took into account potential nucleotide bias caused by PCR and sequencing, and PCR products were purified and directly sequenced on the one hand and sequenced after cloning on the other (>10 clones per sample typically but >20 clones for *P. minimum*). Only sequences containing three or more mutations or those in which the same mutation occurred in more than two clones was accepted as a true sequence variant. Polymorphism analysis and Tajima's *D*-test for neutral mutation were conducted using DnaSP V 5.10.1 (Librado and Rozas, 2009).

A set of online programs was used to characterize the deduced protein as previously reported (Lin et al., 2011). Briefly, the signal peptide was predicted using SignalP V4.1 (Petersen et al., 2011). GPI-SOM (Fankhauser and Mäser, 2005) and PredGPI (Pierlenoi et al., 2008) were used to locate the GPI (glycosylphosphatidylinositol) anchor in the deduced protein sequence. The subcellular localization of AP was predicted using Cello (Yu et al., 2006) with the prediction in highest score presented in Table 1.

### Phylogenetic analysis

Comparisons of amino acid sequences were performed between Dino-APs (alkaline phosphatase of dinoflagellates) and all reported phosphatases, including PhoA^EC^, PhoD, PhoX, phosphodiesterase, and other putatively assigned phosphatase genes identified in marine phytoplankton. Dino-APs were subjected to BLAST analysis against the GenBank, JGI (Joint Genome Institute) genome databases and a comprehensive algal EST datasets collected by our research group to retrieve potential homologs from marine phytoplankton. To examine the phylogenetic relationships between the Dino-APs and counterparts from other organisms, the deduced amino acid sequences from each strain were aligned with top hits in the BLAST analysis and other sequences from representative organisms. The alignment was run using MUSCLE (Edgar, 2004) and manually corrected. Conserved motifs were identified based on the sequence alignment and presented using WebLogo V3 (Crooks et al., 2004). Phylogenetic tree reconstruction was performed using Maximum-Likelihood (Guindon et al., 2010) and Neighbor-joining (NJ) with 1000 bootstraps on the Seaview V4.5.3 platform (Gouy et al., 2010). Bayesian analysis (Hueslenbeck and Ronquist, 2001) was carried out for 3,000,000 generations with burn-in at setting 250,000 (two independent runs were performed and the final standard deviation of split frequencies was lower than 0.01).

## Results

### Identification of introns in APs of *Karenia mikimotoi* and *Prorocentrum minimum*

**Figure 1:**
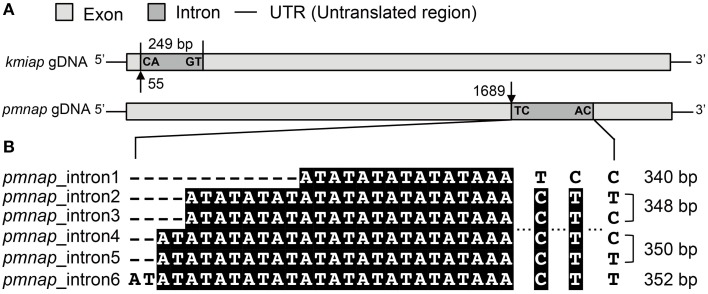
**Analysis of AP introns**. **(A)** Schematic of the intron location and boundary sequences in *kmiap* and *pmnap*, the characters marked at the boundary of the intron represent donor site (left) and acceptor site (right), and the numbers with arrows indicate the nucleotide position of intron from the start codon. **(B)** Sequence comparison of six different variants of intron in *pmnap*, and the length for each intron variant was marked at the right.

To examine the possible existence of introns, AP genes were amplified both from genomic DNA (gDNA) and cDNA from at least one representative strain in each family. Among the eight strains with both gDNA and cDNA analyzed, only APs in *K. mikimotoi* C32-HK (*kmiap*) and *P. minimum* CCMA 15 (*pmnap*) were found to contain introns (Table 1). The *kmiap* intron has CA as the donor site and GT as the acceptor site, while in *pmnap*, the donor site is TC and the acceptor site is AC (Figure 1A). By comparing the introns in *pmnap* and *kmiap*, no noticeable sequence similarity was identified. Unexpectedly, the intron in *kmiap* is translatable, encoding a peptide comprising 83 amino acids that had no BLASTP hits in GenBank. Moreover, at least six different variants of the introns were identified in *pmnap* based on the total of 21 full-length gDNA sequences aligned with the 28 full-length cDNA sequences (Figure 1B). The six intron variants with different lengths were classified by the combination of the repeat numbers of a (AT)n region and three sites of nucleotide substitution (T/C).

### Nucleotide substitution and apparent pseudogenization of AP genes in *Prorocentrum* spp.

**Figure 2:**
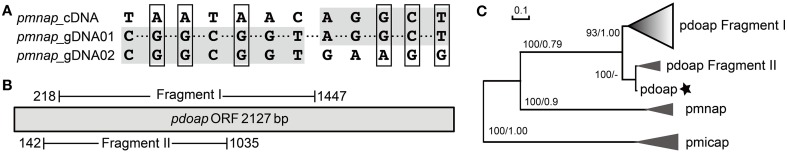
**Sequence comparison of AP genes in *Prorocentrum***. **(A)** Nucleotide substitution of *pmnap* identified between gDNA and cDNA clones, shaded region represents sharing the same nucleotides and box indicates the nonsynonymous substitution. **(B)** Schematic of two different genomic fragments of *pdoap*. **(C)** Phylogenetic tree inferred from nucleotide sequences (common region) of AP gene in *Prorocentrum*. Prodo, *P. donghaiense*; Promini, *P. minimum*, support nodes shown as NJ/ML. Gradient shaded triangles represent the presence of pseudogenes and the asterisk represents the assembled *pdoap* ORF from transcriptomic data (intact tree refered to Supplementary Figure 1B).

By comparing full-length AP ORFs derived from cDNA (28 clones) with gDNA (21 clones), we found sequence discrepancies between the cDNA and the gDNA in *P. minimum*. This indicated either mRNA editing or differential recovery of paralogous sequences from the genomic DNA and the cDNA samples. In *P. minimum*, we identified only one type of ORF in *pmnap* cDNA while two different types in gDNA, with nucleotide substitutions observed in a total of 12 sites (Figure 2A, Supplementary Table 2). Except for a pyrimidine transitional substitution at one site, all the sites had transversional replacement. Half of those substitutions were synonymous. A 1463 bp AP gene fragment was obtained from *P*. *micans* genomic DNA, which also possessed multiple nucleotide substitutions. All of these 13 clone sequences could be translated into partial AP proteins of 487 amino acids in length, which can be grouped into five different variants resulting from 143 nucleotide substitutions (Supplementary Figure 1A).

A predicted full-length contig of the AP gene (*pdoap*) was assembled from a transcriptome dataset of *P. donghaiense* (Shi et al. unpublished data). The length of the deduced protein PDOAP was close to the PMNAP in *P*. *minimum* and the pairwise comparison between these two protein sequences showed 100% coverage, 87% identity, and *E*-value 0.0. However, PCR with different primer combinations consistently led to the recovery of two fragments from *P. donghaiense* genomic DNA, respectively sharing high similarity to two separate parts of *pdoap*. Fragment I was acquired using the specific primer pair prodoAPgF2/gR2 (32 clones) (Supplementary Table 1), and Fragment II using the Dino-AP degenerate primer pair DinoAPNF4b/NR6 (10 clones) (Figure 2B). Some of the Fragment I sequences (25 out of a total of 32 clones) appeared to be pseudogenes because they could not be translated into contiguous peptides due to one or more premature stop codons caused by nucleotide substitution/insertion, while the predicted peptide of Fragment II covered 57% of the predicted PDOAP with *E*-value 0.0. A phylogenetic tree inferred from the common nucleotide sequences shared by the assembled *pdoap, pdoap* fragments, *pmnap*, and *pmicap* showed that *pdoap* grouped together with *pmnap*, but distant from *pmicap* (Figure 2C, Supplementary Figure 1B). Moreover, *pdoap* fragment I dominated by pseudogene clones formed a separate branch from translatable fragment II and the assembled *pdoap*. All these results suggest that gene pseudogenization occurred after divergence of *P. donghaiense* and *P. minimum*.

### High AP gene sequence diversity in *Alexandrium* spp.

To explore the conservation of AP gene sequences within a species, specific primers pairs AlexAPFLF/R (Supplementary Table 1) designed previously based on the 5′ and 3′ UTR region in *A. catenella* (ACHK-NT) (Lin et al., 2012a) were used to acquire the full length AP ORFs from three different *Alexandrium* species, including four additional strains from *A. catenella* (18S Clade IIC/28S Group IV), and one strain each from *A. tamarense* (18S Clade IIB/28S Group III) and *A. fundyense* (18S Clade I/28S Group I) (Table 1) (Wang et al., 2014). We successfully retrieved the complete ORFs from most of the tested *Alexandrium* spp.; however, the full-length AP gene in the strain *A. tamarense* CCAP1119/1 was obtained only by using specific primer pairs CCAP1119/1 APFLF/R designed based on the UTR region acquired as described in methodology (Supplementary Table 1).

In *A. catenella*, strain ACHK-NT and the other three strains (ACHK-T, ATDH02, and ATMJ01) share the same gene sequence (*alecaap*). Strain ATCI01 was the only exception in which a non-synonymous mutation occurred in 23 clones (variant I), and another five different AP variants occurred in one clone each (Table 2, Supplementary Table 3). Sequence comparison of the six different variants showed high nucleotide polymorphisms scattered along the whole length of the ORF region. Besides a 12-bp insertion, which was detected only in variant VI, there were 99 SNP sites, only one third of them being synonymous mutations (Table 2, Supplementary Table 3). Based on the nucleotide sequences of the six different variants, Tajima's *D*-value was calculated using DnaSP, which gave a value of 0.26 (*p* > 0.10), suggesting that those polymorphic sites were neutral mutations (Tajima, 1989). All the AP protein variants from *A*. *catenella* were predicted to be extracellular (Table 1).

**Table 2:** **Summary of nucleotide polymorphism in *Alexandrium* spp**.

**Strain**	**nt variants**	**aa variants**	**Deletion (bp)**	**Total SNP sites (Syn/Nonsyn)**	**Tajima's D**
*Alexandrium catenella* ATCI01	6 (gDNA)	6	12	99 (34/65)	0.26 (*P* > 0.10)
*Alexandrium tamarense* CCAP1119/1	2/37 (gDNA/cDNA)	36	12/24/129	139 (39/100)	−2.75 (*P* < 0.001)
*Alexandrium fundyense* CCMP1719	6/18 (gDNA/cDNA)	13	24/30/42	9 (3/6)	2.46 (*P* < 0.05)

*nt, nucleotide; aa, amino acid*.

**Figure 3:**
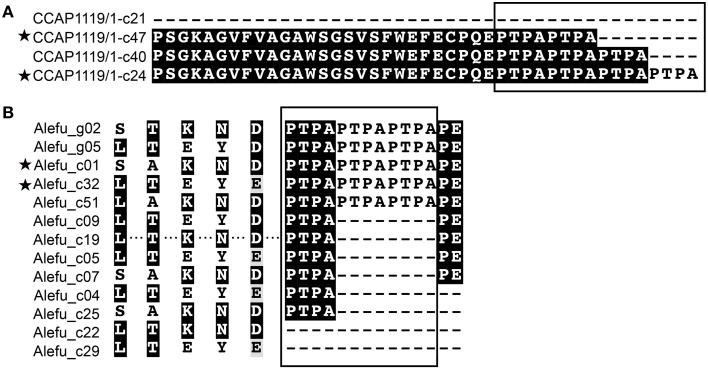
**Sequence comparison showing intraspecific AP polymorphism and differences between closely related species of *Alexandrium***. **(A)** *A. tamarense* (strain CCAP1119/1); **(B)** “Alefu” represents *A. fundyense* (strain CCMP1719). The boxes mark the “PTPA” repeat motif with different repeat numbers in different variants. Asterisks indicate that this variant was present in both gDNA and cDNA clones.

In strain *A. tamarense* CCAP1119/1, among the 83 full-length clones we sequenced (18 for gDNA, 65 from cDNA), 139 nucleotide polymorphic sites and deletions in both gDNA and cDNA clones were observed, making the *ataap* highly polymorphic (Table 2, Figure 3A). There were three variants of deletions (12, 24, and 129 bp) when compared with the longest complete ORF (2184 bp) which was also the dominant variant in both gDNA and cDNA clone libraries. Overall 36 different ORF variants were identified considering both the gDNA and cDNA amplicons together. We noticed that the changes of amino acid sequences in different variants caused by 12 and 24 bp deletions resulted in the loss of different numbers of the “PTPA” repeat unit, which was completely absent in the variant with 129-bp deletions (Figure 3A). Correspondingly, the variants detected in CCAP1119/1 were predicted to have potentially different subcellular localization, extracellular in most cases but also cytoplasmic and chloroplast, which was exceptional compared with all other APs in *Alexandrium* spp. we tested (Table 1). With all the variants considered, the Tajima's *D*-value was −2.75 (*p* < 0.001), indicating that this gene has evolved under directional selection (Tajima, 1989).

In strain *A*. *fundyense* CCMP1719, a total of 15 gDNA and 53 cDNA full-length clones were obtained, also exhibiting highly polymorphic gene sequences. We identified six variants of *afuap* in gDNA clones and 18 variants in cDNA clones, only two of which were shared by both, which comprise 13 variants of protein sequences in total containing the “PTPA” repeat unit in different numbers as well (Figure 3B). The corresponding subcellular localization of all AFUAP variants was predicted to be in the plasma membrane, appearing to be unique among all detected dinoflagellate strains. Moreover, different from the result in ATCI01 and CCAP1119/1, the Tajima's D neutral test for *alefuap* was 2.46 (*p* < 0.05), suggesting evolution by balancing selection (Table 2).

### Classification of dinoflagellate APs as an atypical AP (PhoA^aty^)

From the full-length AP genes from 10 dinoflagellate strains (including ORF fragments from *P*. *micans*) obtained in this study, and a predicted AP full-length ORF region retrieved from the transcriptome of *P*. *donghaiense* (Shi et al. unpublished) and the genome of *S*. *kawagutii* respectively (Lin et al. unpublished), in total we collected AP gene sequences from 15 dinoflagellate strains (three of them have been published in Lin et al., 2011, 2012a,b), representing four core dinoflagellate families (Table 1). Overall, all the deduced dinoflagellate AP proteins (Dino-APs) shared similar chemical characteristics and predicted protein structures, e.g., possessing a signal peptide and GPI anchor (Table 1), with that from *S. kawagutii* being the only exception.

**Figure 4:**
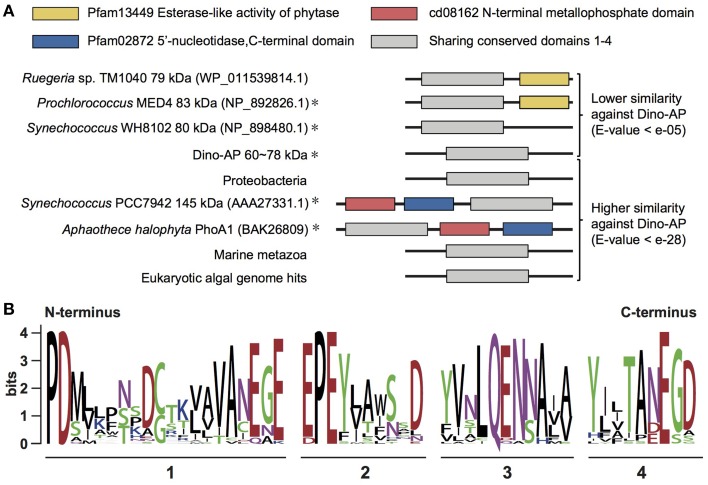
**Comparative structural analysis of AP in different organisms**. **(A)** Sequence comparison of PhoA^aty^ among different organisms and the section containing four conserved domains (adapted from Moore et al., 2005 and Scanlan et al., 2009). Representative sequence labeled with “asterisk” indicated this gene has been characterized partially. **(B)** WebLogo showing the consensus of amino acid sequences acquired from the alignment of four conserved domains (detail alignment shown in Supplementary Figure 2).

To determine which of the currently classified types of AP our Dino-APs belong to, pairwise sequence comparisons were carried out between the Dino-APs and all reported phosphatases. Overall, BLAST analysis results of Dino-APs revealed no recognizable functional domains in the sequences. The similarity between Dino-APs and typical PhoA^EC^ was too low to identify the well-characterized PhoA^EC^ domains in Dino-APs. Further comparison showed that Dino-APs only shared sequence similarity with partial sequences of a group of putative phosphatases identified in cyanobacteria (Figure 4A). BLASTP of Dino-APs against GenBank non-redundant protein database showed that the top100 hits (*e*-value < e^−28^) included three major groups, eukaryotic algae, marine animals, and bacteria (cyanobacteria and proteobacteria).

Despite the low sequence similarity among those putative APs (*E*-value e^−05^ to e^−28^), we were able to identify four conserved motifs from the amino acid alignment of all the AP gene sequences from dinoflagellates and other organisms (Figure 4B, Supplementary Figure 2). Although none of these four motifs fit any previously characterized domain data, there is a significant conservation of aspartic acid (D) and glutamic acid residues (E) in those motifs. Based on the presence of the common motifs in these APs, some of which have previously been assigned as an atypical PhoA type, we were led to classify these putative APs as PhoA^aty^ to distinguish them from the typical PhoA^EC^ and other phosphatases.

### Phylogenetic analysis of phoA^aty^

**Figure 5:**
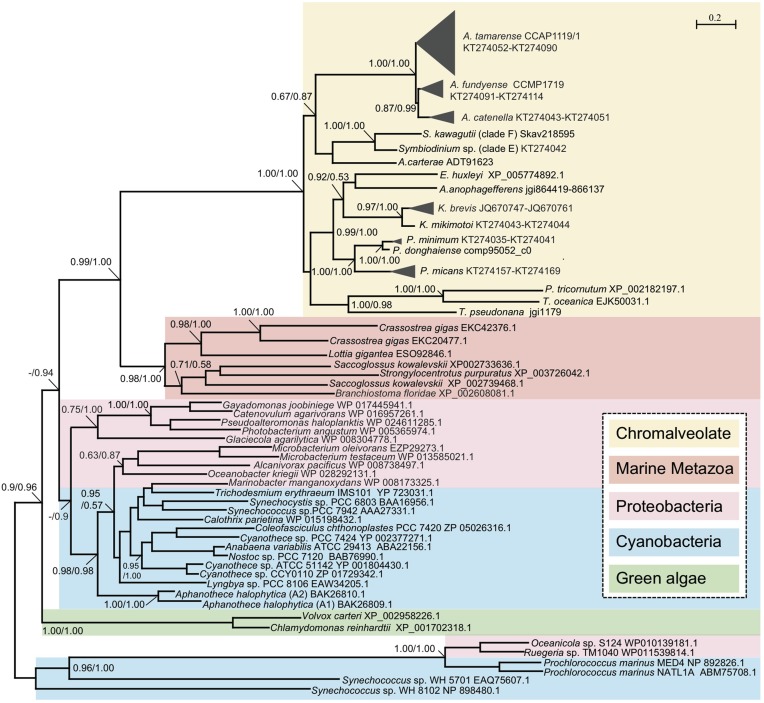
**Phylogenetic tree inferred from amino acid sequences of PhoA^aty^ in marine organisms**. Tree topology shown as inferred from PhyML (Dayhoff model), and Bayesian analysis (BE) was also carried out based on Dayhoff model with similar tree topology. Support of nodes for most branches are shown as SH-like (Shimodaira-Hasegawa-like procedure) from ML and Bayesian posterior probability (ML/BE).

To gain an evolutionary perspective of these PhoA^aty^ proteins, a phylogenetic tree was inferred based on the amino acid alignment of AP (unique variants) from dinoflagellates and representative species of other organisms. As shown in Figure 5, APs from cyanobacteria and proteobacteria formed a specific clade. With this clade as the root, two freshwater green algae formed a clade, clearly separated from a large clade comprising PhoA^aty^ identified from dinoflagellates and other chromophytes (Chromalveolate clade), marine metazoans and bacteria. In the latter clade, APs from marine metazoans and chromalveolate respectively, formed monophyletic groups sister to each other, while cyanobacteria and proteobacteria inter-mingled as a separate group. In the Chromalveolate PhoA^aty^ clade, diatoms formed a monophyletic lineage while dinoflagellates appeared to be paraphyletic due to the placement of the diatom group that separated AP of the *Prorocentrum*/*Karenia* clade from that of the *Alexandrium*/*Amphidinium*/*Symbiodinium* clade and the alliance of haptophytes and pelagophytes that separated the subclade of *Karenia* spp. from that of *Prorocentrum* spp. Within the dinoflagellate APs clusters, each genus (e.g., *Alexandrium*) formed a monophyletic clade and all variants in each species formed a monophyletic group. A similar tree topology for APs of *Prorocentrum* spp. was observed between the amino acid and nucleotide sequences (Figure 2C, Supplementary Figure 1).

## Discussion

### Unusual introns and within-lineage duplication in dino-APs evolution

In contrast to the long-held notion that dinoflagellate genes have few introns (for review see Lin, 2011), fairly abundant introns have recently been reported in dinoflagellates (Bachvaroff and Place, 2008; Shoguchi et al., 2013). In this study, we detected introns in the AP gene in two out of the 12 dinoflagellates we examined, *kmiap* and *pmnap*, but their splice sites were unconventional. The translatable nature of the *kmiap* intron suggests that it may be functionally similar to Group II introns, a class of self-catalytic ribozymes containing an internal ORF which is capable of its own excision from precursor RNAs. This type of intron has been found only in bacteria and organellar genomes of fungi, plants, and protists (Rodríguez-Trelles et al., 2006; Roy and Gilbert, 2006).

Gene duplication is the major driving force in the evolution of functional diversification, and the duplicated genes (paralogs) can maintain the function of its parental gene, lose function due to dramatic sequence mutation (pseudogenization), perform partial function of the parental gene (subfunctionalization), or acquire and preserve a novel function (neofunctionalization) due to significant sequence divergence (Lynch and Conery, 2000; Zhang, 2003). The many variants of APs in each of the dinoflagellate strains examined in this study are a clear indication of gene duplication. The fact that the variants are grouped by species in the phylogenetic analyses further indicates that gene duplication occurs within a species. Given that *Alexandrium* is the latest-diverging genus of dinoflagellates (John et al., 2003), this result suggests that some of the gene duplications of Dino-APs might be quite recent. The clustering of pseudogenes in *P. donghaiense* suggests pseudogenization in the process or soon after gene duplication of Dino-APs. Moreover, the high sequence similarity with functional gene homologs (except for the internal stop codons or frame shifts), as is the case in *P. donghaiense*, suggests that these are young pseudogenes (Zhang, 2003).

Human AP is considered a multigene family derived from an original AP gene by gene duplication (Weiss et al., 1988). Comparably, multicopies of AP also occur in dinoflagellates and their monophyletic grouping within each species in the phylogenetic tree also suggests that these copies have also descended from a common ancestral gene. To describe the evolution of a multigene family, two different models have been proposed, concerted evolution and birth-and-death evolution (Nei and Rooney, 2005). In concerted evolution, paralogs of a gene within one species are closer to each other than to those in another species, even though gene duplication might have occurred before speciation. The monophyletic grouping of AP paralogs by species observed in Dino-APs is consistent with this model. However, the occurrence of pseudogenes and recently duplicated paralogs in dinoflagellate APs makes it fit the birth-and-death model better. In the birth-and-death model, new genes are generated by duplication, but subsequently whereas some of these will stay in the genome others are subject to deletion or inactivation during evolution. Therefore, most gene families that produce variable gene products are subject to birth-and-death evolution (Nei and Rooney, 2005). This model has also been invoked to explain the evolution of actin in dinoflagellates (Kim et al., 2011).

### Sequence divergence of dino-APs and ecological implication

Duplicated genes may diverge and undergo functional differentiation (Kondrashov et al., 2002; Zhang, 2003). As the prototype AP, PhoA^EC^ is an enzyme with promiscuous substrate specificity, which has a selective advantage during gene duplication to optimize the specificity and activity for a diverse type of substrates (Yoshikuni et al., 2006; Zalatan et al., 2006). Moreover, both PhoX and PhoD in bacteria exhibit promiscuous enzymatic activities on phosphomonoesters and phosphodiesters (Luo et al., 2009; Sebastian and Ammerman, 2011). It has been well documented that dinoflagellates are able to utilize different types of DOP, each as a sole P source (Huang et al., 2005; Oh et al., 2010), suggesting that Dino-APs are also promiscuous. Substrate promiscuity has clear advantages for versatility in acquiring P from different forms of DOP, and might be an evolutionary adaptation to the abundant and chemically diverse DOP in the ocean (Paytan and McLaughlin, 2007).

It is interesting to observe that the AP genes of different *Alexandrium catenella* strains (most of them isolated from East China Sea) share the same sequence, except that in strain ATCI01, which was isolated from the South China Sea (Wang et al., 2014). *A. tamarense* (strain CCAP1119-1 from England) and *A. fundyense* (strain CCMP1719 from the Gulf of Maine) exhibit high AP sequence variations. The Tajima' *D*-values for these AP sequences were totally different in these three geographic regions (China Seas, Bay of Tamar Esturary, England, and the Gulf of Maine, USA), which indicate that the evolution of AP in *Alexandrium* spp. is probably driven by habitat-specific environmental conditions. As an example, it has been thought that the independent loss and duplication of the intestinal AP in vertebrate lineages are driven by their respective substrate landscapes dictated by their different gut microbiomes and diets (Yang et al., 2012). Furthermore, our results also showed that deduced protein variants in *Alexandrium* spp. might have differential subcellular localizations as computationally predicted, *A*. *catenella* in the extracellular space, *A*. *fundyense* in the plasma membrane, and *A*. *tamarense* in three possible subcellular compartments. These differential subcellular distributions of APs would enable the different dinoflagellate species to utilize different DOP sources (e.g., intracellular and extracellular). Therefore, the diverging sequences of different Dino-AP potentially have significant ecological implications (e.g., utilizing different sources of DOP) (Lin et al., 2012a).

### PhoA^aty^ as a phylogenetically distinct type of AP in marine microorganisms

Sequence comparisons and phylogenetic analyses of Dino-APs indicated that they comprise a type of AP–PhoA^aty^ similar to those reported in several bacteria. Although there is not yet sufficient biochemical data of PhoA^aty^ (i.e., metal ion requirement, substrate specificity) for us to ascribe the observed sequence divergence to potential enzymatic structural and functional diversification, we can obtain some insights from the relatively well-characterized counterparts. As confirmed in PhoA^EC^, the metal-binding site shows a preference for histidine (H) and aspartic acid residues (D), consistent with what has been observed in most Zn-binding proteins (Galperin and Jedrzejas, 2001; Patel et al., 2007). PhoA^aty^ in different species shares conserved motifs, in which there is a significant conservation of aspartic acid (D) and glutamic acid residues (E) that have been found to be responsible for Ca-binding in PhoX. Besides, a highly conserved Ca-binding “proline-aspartic acid” motif is shared by PhoA^aty^, PhoX, and phosphotriesterase (Wu et al., 2007; Zaheer et al., 2009; Yong et al., 2014).

The high sequence divergence observed in PhoA^aty^ may be attributed to serial gene duplications followed by divergent evolution between each species. As observed in the phylogenetic analyses (Figure 5), PhoA^aty^ in green algae was placed in a separate cluster from the other eukaryotic algae, and the PhoA^aty^ from dinoflagellates, haptophytes, and stramenopiles were all mingled together. This coincides with the different endosymbiosis histories of these algae: green algae emerged from primary endosymbiosis and the rest from secondary endosymbiosis or tertiary replacement (Falkowski et al., 2004). It is interesting to note that AP from *Karenia* spp. is close to that from *E*. *huxleyi* while the plastid of *Karenia* and other Kareniacea lineages arose as a result of tertiary replacement from a haptophyte origin (Keeling et al., 2004; Tirichine and Bowler, 2011; Jackson et al., 2013). HGT has been considered as a significant driver of gene innovation in both dinoflagellates (Wisecaver et al., 2013) and diatoms (Bowler et al., 2008). Based on both the sequence comparison and phylogenetic analysis, PhoA^aty^ in the chromalveolate algae likely arose through HGT from a common ancestor. Similarly, it has been suggested that PhoX has undergone horizontal gene transfer among different marine bacteria (Zaheer et al., 2009; Kathuria and Martiny, 2011).

The remarkably low sequence similarity between the identified multiple types of APs in different algal lineages qualifies this group of enzymes as Non-homologous ISofunctional Enzymes (NISE), which refers to enzymes that lack detectable sequence similarity but catalyze the same biochemical reactions (Omelchenko et al., 2010). The pressure of adapting to the fluctuating nutrient availability in marine environments is likely to trigger the convergent evolution of a group of NISE toward APs to hydrolyze phosphoesters from different ancestral genes. Over-representation of NISE, such as in the case of antioxidant analogous enzymes, may imply a powerful selective pressure from rapid oxygenation, since the best-known superoxide dismutase (SOD) enzymes include four distinct structural types with different metal ion requirements (Omelchenko et al., 2010). Similarly, multiple types of APs also exhibit different metal requirements, with most characterized APs in marine microorganisms apparently having adopted Ca as a cofactor instead of a typical Zn/Mg type (Luo et al., 2009; Sun et al., 2012; Lin et al., 2013). The adoption of Ca may simultaneously require Fe as the cofactor partner, as found in PhoX (Yong et al., 2014). Although whether Ca/Fe is the only pair that replaces Zn/Mg remains to be further studied, the differential divalent metal cation requirements of multiple types of APs represents an interesting case of adaptive evolution in organisms living in the oceanic habitat where Zn bioavailability is often limited (Sebastián and Ammerman, 2009; Kathuria and Martiny, 2011).

## Concluding remarks

In this study, we found introns, pseudogenes, and presence of multiple copies of AP in dinoflagellates indicating that the evolution of APs in dinoflagellates has been driven by gene duplication and fits the birth-and-death model. The Tajima's *D*-value differed amongst the three geographic species of *Alexandrium* predictive of differential subcellular localizations amongst the various deduced proteins, implying APs in these species have evolved under different environmental selection pressure. Phylogenetic analysis showed that Dino-APs, as well as putative APs from other eukaryotic phytoplankton, might be classified as an atypical type of AP, PhoA^aty^, which is prevalent in marine microorganisms and shares a common ancestral gene. We found evidence that environmental stress and endosymbiosis (plastid acquisition) might have contributed to the divergent evolution of PhoA^aty^. On this basis, we propose that multiple types of APs in marine ecosystems could be considered a group of NISE, sharing weak sequence similarity and different metal ion requirements while performing the same phosphoester hydrolyzing function. Adaption to the heterogeneous and limited availability of metal ions and phosphate in the ocean (Moore et al., 2013), by owning multiple types of AP would provide a competitive advantage to marine phytoplankton living in different environments.

### Conflict of interest statement

The authors declare that the research was conducted in the absence of any commercial or financial relationships that could be construed as a potential conflict of interest.

## Abbreviations

AP: alkaline phosphatase
Dino-AP: dinoflagellate AP
PhoA^EC^: typical *E*. *coli*-type AP
PhoA^aty^: atypical type of PhoA.
