# Supplementary material for: Rapidly diverging evolution of an atypical alkaline phosphatase (PhoAaty) in marine phytoplankton: insights from dinoflagellate alkaline phosphatases
Source: Front Microbiol. 2015 Aug 25;6:868. doi: 10.3389/fmicb.2015.00868 (PMC4548154; doi:10.3389/fmicb.2015.00868)
Supplement: Supplementary file 1 [file Table1.PDF]

Supplementary Table 1. Primers used in this study.

Applications	Primer name	Primer direction	Primer sequence (5'-3')
cDNA synthesis for all species	454BT7-dT	R	GAGACTATGCGCCTTGCCAGCCCGCTCAGTAATACGACTCACTATAGGGAGTTTTTTTTTTTTTTTTVN
AP PCR for all species	DinoAPNF4a	F	TTTGGWATGGGWGCMGCRGARCA
	DinoAPNF4b		GGTATGGGWGCMGCRGARCAGATT
	DinoAPNR6	R (outward-inward)	CTTRTCRTCRCCTTCRTYMGCKGT
	DinoAPNR7a		CTGCTTCTCTGTGGCWGTCACTG
	DinoAPNR7b		CCCYGCYGTSCCCTGCTTCTCTGT
A. carterae CCMP1314	AmpAPFLF1	F	CCCTGTAGAGAGAAGATCATCCACAGAAGACCAATTATG
	AmpAPFLR1	R	AAAAGTGAAGCGGCTCTATGTACGACCGTTCA
K. mikimotoi C32-HK	KarmiFLF1	F	GCATCAAGCTCAGCAGYAAGTARACC
	KarmiFLF2		AGCRCAGCACTTTCCAGCTTCAGC
P. minimum CCMA15	ProminiAPFLF1	F	TCAGTCGGTCACGACGCCCGCTCAC
	ProminiAPFLF2		TCACTCCAGACAGTGAATCGCTCCAG
	ProminiAPFLR1	R	TCGAGAGGTGATGTACATGAGAGGC
	ProminiAPFLR2		AACTATGGCACTGGAGGCTCTCC
P. donghaiense (pseudogne)	ProdoAPgF2	F	TTCACGTCATCGATTACGACGATGC
	ProdoAPgR2	R	TCGTCGTCCGAGCTGTTGTACAGC
Symbiodinium sp.	SymbioAPFLF1	F	TGAGATTGGCTCAAGGACAGCCGTGC
	SymbioAPFLF2		AAGGACAGCCGTGCATCTGACGC
	SymbioAPFLR1	R	CGTAGCCACGGTGCCGCACAACAGAA
	SymbioAPFLR2		TCGCTCCACGGACCTTCCAGAAACTTGGG
A. catenella (ACHK-NT, ATMJ, ATDH02, ATCI01) *A.fundyense* CCMP1719	AlexAPFLF1	F	GTAGTCTCCACGAGCCACSCTCCAGRCGTT
	AlexAPFLF2		TCCAGRCGTTYGTCRCCAGCCTGTCCCTGCCATG
	AlexAPFLR1	R	TGTCTCTGCTCCTCCTRTRCGG
	AlexAPFLR2		AACCKTTGCCACGGTCCTGRC
A. tamarense CCAP1119/1	CCAP1119/1APFLF1	F	TTGTTGTCTCCACGAGCCACGCTC
	CCAP1119/1APFLF2		CGTTCGTCACCAGCCTGTCCCTG
	CCAP1119/1APFLF3		TCACCAGCCTGTCCCTGCCATG
	CCAP1119/1APFLR1	R	AACCAACTTGAGACGGGATGTAAGACGAAATTCC
	CCAP1119/1APFLR2		ACACAATGTACTTGAAGGGGTAGGTCGC
	CCAP1119/1APFLR3		TGGAATTAAATCCAATGTGAGACGAGAGG
