# Supplementary material for: Rapidly diverging evolution of an atypical alkaline phosphatase (PhoAaty) in marine phytoplankton: insights from dinoflagellate alkaline phosphatases
Source: Front Microbiol. 2015 Aug 25;6:868. doi: 10.3389/fmicb.2015.00868 (PMC4548154; doi:10.3389/fmicb.2015.00868)
Supplement: Supplementary file 2 [file Table2.PDF]

Supplementary Table 2. Nucleotide substitution in *pmnap*.

| Number | Site (gDNA / cDNA) | nt (gDNA) | nt (cDNA) | Site (AA) | AA (gDNA) | AA (cDNA) |
|---|---|---|---|---|---|---|
| 1 | 879 | C | T | 293 | V | V |
| 2 | 883 | G | A | 295 | D | N |
| 3 | 1103 | G | A | 368 | G | D |
| 4 | 1107 | C | T | 369 | N | N |
| 5 | 1147 | G | A | 383 | G | S |
| 6 | 1263 | G | A | 421 | G | G |
| 7 | 1461 | T | C | 487 | G | G |
| 8 | 2170/1818 | G/A | A | 606 | P | P |
| 9 | 2326/1974 | A/G | G | 658 | A | A |
| 10 | 2354/2002 | A/G | G | 668 | T/A | A |
| 11 | 2406/2054 | G/C | C | 685 | S/T | T |
| 12 | 2429/2077 | G/T | T | 693 | G/W | W |
