# Supplementary material for: Rapidly diverging evolution of an atypical alkaline phosphatase (PhoAaty) in marine phytoplankton: insights from dinoflagellate alkaline phosphatases
Source: Front Microbiol. 2015 Aug 25;6:868. doi: 10.3389/fmicb.2015.00868 (PMC4548154; doi:10.3389/fmicb.2015.00868)
Supplement: Supplementary file 3 [file Table3.PDF]

Supplementary Table 3. Summary of nucleotide polymorphism in different variants of *ATCI01-ap*.

Site (nt/aa)	Variant I (23)	Variant II (1)	Variant III (1)	Variant IV (1)	Variant V (1)	Variant VI (1)
656 /219	G /G	A /D	G /G	G /G	G /G	G /G
662 /221	C /A	T /V	C /A	C /A	C /A	C /A
700 S	C	T	C	C	C	C
736 /246	C /L	A /I	C /L	C /L	C /L	C /L
756 S	G	T	G	G	G	G
769	A	C	A	A	A	A
770 /257	A /N	G /R	A /N	A /N	A /N	A /N
810 S	G	T	G	G	G	G
812 /271	G /G	A /D	G /G	G /G	G /G	G /G
815 /272	T /L	C /P	T /L	T /L	T /L	T /L
824 /275	A /D	G /G	A /D	A /D	A /D	A /D
836 /279	C /A	A /D	C /A	C /A	C /A	C /A
885 S	T	C	T	T	T	T
912 S	C	T	T	C	C	C
921 /307	G /E	T /D	T /D	G /E	G /E	G /E
927 S	C	T	T	C	C	C
930 S	G	A	A	G	G	G
940 S	C	T	T	C	C	C
946 /316	A /I	G /V	G /V	A /I	A /I	A /I
973 /325	T /S	G /A	G /A	T /S	T /S	T /S
978 S	T	C	C	T	T	T
1011 S	C	T	T	C	C	C
1038 S	C	T	T	C	C	C
1050 /350	G /E	T /D	T /D	G /E	G /E	G /E
1071 S	C	T	T	C	C	C
1097 /366	C /A	T /V	T /V	C /A	C /A	C /A
1111 /371	G /D	A /N	A /N	G /D	G /D	G /D
1133 /378	C /A	T /V	T /V	C /A	C /A	C /A
1150 /384	A /K	C /Q	C /Q	A /K	A /K	A /K
1163 /388	A /D	G /G	G /G	A /D	A /D	A /D
1169 /390	A /E	C /A	C /A	A /E	A /E	A /E
1190 /397	A /D	G /G	G /G	A /D	A /D	A /D
1211 /404	T /I	A /N	A /N	A /N	T /I	T /I
1225 /409	G /D	A /N	A /N	A /N	G /D	G /D
1253 /418	C /A	T /V	T /V	T /V	C /A	C /A
1334 /445	G /G	T /V	T /V	T /V	G /G	G /G
1339 /447	A /T	T /S	T /S	T /S	A /T	A /T
1343 /448	C /A	T /V	T /V	T /V	C /A	C /A
1352 /451	A /Y	G /C	G /C	G /C	A /Y	A /Y
1369 /457	C /P	G /A	G /A	G /A	C /P	C /P
1381 /461	A /K	C /Q	C /Q	C /Q	A /K	A /K
1394 /465	T /F	C /S	C /S	C /S	T /F	T /F
1410 S	T	C	C	C	T	T
1430 /477	C /S	C /S	G /C	G /C	C /S	C /S
1455 S	G	G	A	A	G	G
1459 /487	T /S	T /S	G /A	G /A	T /S	T /S

1518 S	T	T	C	C	T	T
1522 /508	G /D	G /D	A /N	A /N	G /D	G /D
1560 S	T	T	C	C	T	T
1564 /522	C /L	C /L	T /F	T /F	C /L	C /L
1579 /527	A /K	A /K	A /K	C /Q	A /K	A /K
1582 S	C	C	C	T	C	C
1595 /532	T /I	T /I	T /I	G /S	T /I	T /I
1597 /533	T /L	T /L	T /L	G /V	T /L	T /L
1609 /537	C /Q	C /Q	C /Q	G /E	C /Q	C /Q
1657 /553	G /E	G /E	G /E	A /N	G /E	A /N
1659	G	G	G	T	G	T
1666 /556	G /D	G /D	G /D	C /H	G /D	C /H
1679 /560	C /P	C /P	C /P	T /L	C /P	T /L
1691 /564	C /A	C /A	C /A	C /A	C /A	T /V
1694 /565	C /A	C /A	C /A	C /A	C /A	T /V
1695	C	C	C	C	C	T
1709 /570	C /A	C /A	C /A	C /A	C /A	T /V
1773 /591	T /F	T /F	T /F	T /F	T /F	A /L
1806 S	A	A	A	A	G	G
1837 /613	T /S	T /S	T /S	T /S	C /P	C /P
1855	G	G	G	G	C	C
1857 /619	G /G	G /G	G /G	G /G	C /R	C /R
1866 S	T	T	T	T	C	C
1875 S	C	C	C	C	T	T
1881 S	C	C	C	C	T	T
1908 S	C	C	C	C	A	A
1935 S	C	C	C	C	T	T
1940 /647	A /D	A /D	A /D	A /D	G /G	G /G
1944 S	C	C	C	C	T	T
1961 /654	C /A	C /A	C /A	C /A	T /V	T /V
1983 S	C	C	C	C	T	T
2011	T	T	T	T	C	C
2013 /671	C /F	C /F	C /F	C /F	T /L	T /L
	-	-	-	-	-	2026-2037 12bp nt insertion /4 AA (PEPT)
2028 S	T	T	T	T	G	G(2040)
2030 /677	C /A	C /A	C /A	C /A	A /E	C /A (2042)
2037 S	G	G	G	G	A	G(2049)
2043 S	G	G	G	G	G	A(2055)
2059	G	G	G	G	G	A(2071)
2060 /687	G /G	G /G	G /G	G /G	G /G	C /T (2072)
2063 /688	A /Q	A /Q	A /Q	A /Q	A /Q	C /P (2075)
2067 S	A	A	A	A	A	G(2079)
2071	G	G	G	G	G	A(2083)
2072 /691	G /G	G /G	G /G	G /G	G /G	C /T (2084)
2077 /693	A /T	A /T	A /T	A /T	A /T	G /A (2089)
2086	G	G	G	G	G	C(2098)
2088 /696	C /D	C /D	C /D	C /D	C /D	G /Q (2100)
2090 /697	G /G	G /G	G /G	G /G	G /G	A /D (2102)
2105 /702	A /H	A /H	A /H	A /H	A /H	T /L (2117)
2116 /706	C /L	C /L	C /L	C /L	C /L	T /F (2128)
2121 S	A	A	A	A	A	G(2133)
2122 /708	C /P	C /P	C /P	C /P	C /P	T /S (2134)
2127 S	G	G	G	G	G	A(2139)
2144 /715	G /G	G /G	G /G	G /G	G /G	T /V (2156)

Note: No. in the brackets after "Variant" represent detected clone number; No. in the left column represents the nucleotide site in Variant VI; Shaded area indicate the nucleotide substituions observed insideof one codon.
