# Supplementary figures and images for: Rapidly diverging evolution of an atypical alkaline phosphatase (PhoAaty) in marine phytoplankton: insights from dinoflagellate alkaline phosphatases
Source: Front Microbiol. 2015 Aug 25;6:868. doi: 10.3389/fmicb.2015.00868 (PMC4548154; doi:10.3389/fmicb.2015.00868)

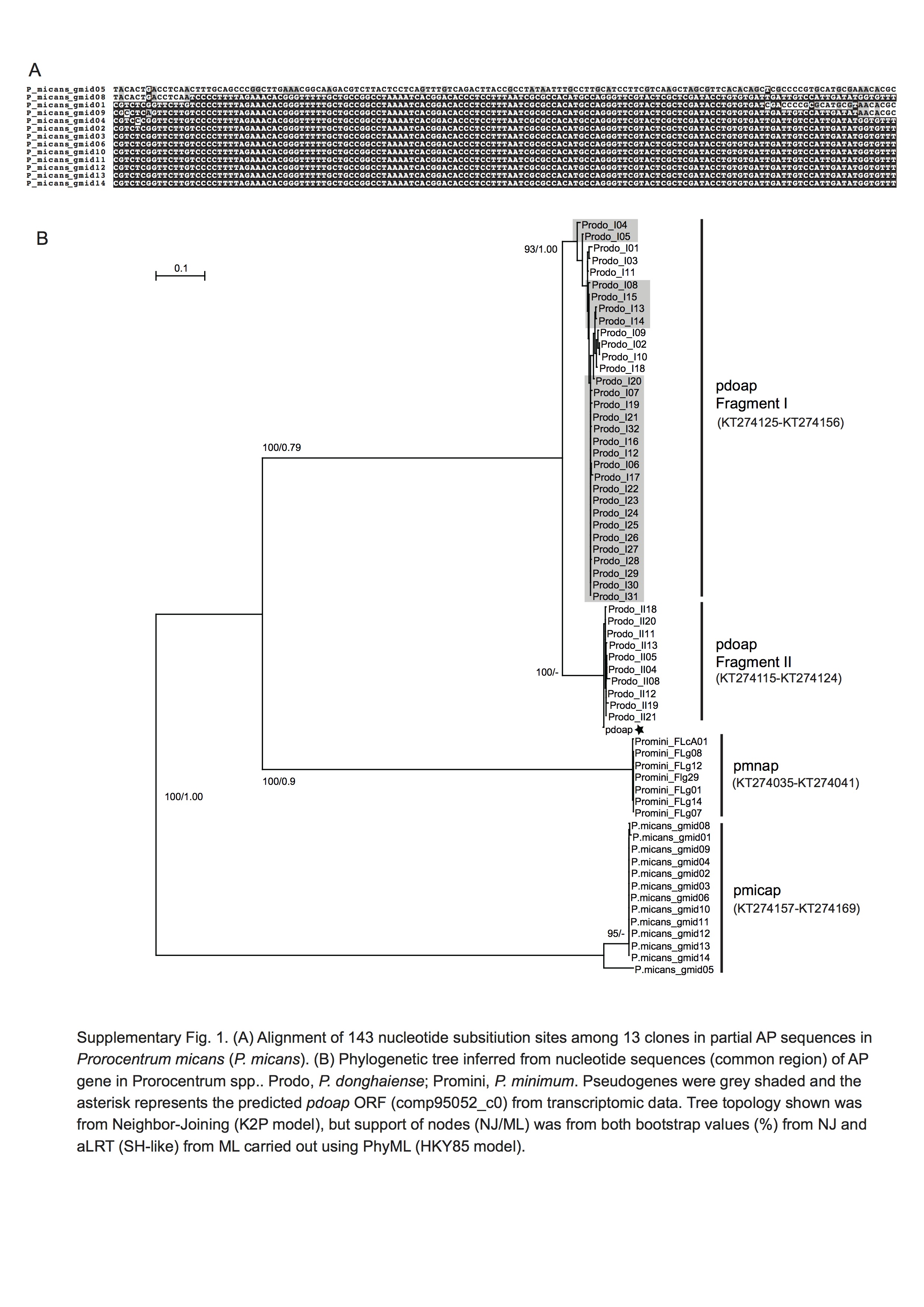

Supplement: Supplementary file 4 [file Image1.JPEG]
