# Supplementary material for: Rapidly diverging evolution of an atypical alkaline phosphatase (PhoAaty) in marine phytoplankton: insights from dinoflagellate alkaline phosphatases
Source: Front Microbiol. 2015 Aug 25;6:868. doi: 10.3389/fmicb.2015.00868 (PMC4548154; doi:10.3389/fmicb.2015.00868)
Supplement: Supplementary file 5 [file Image2.PDF]

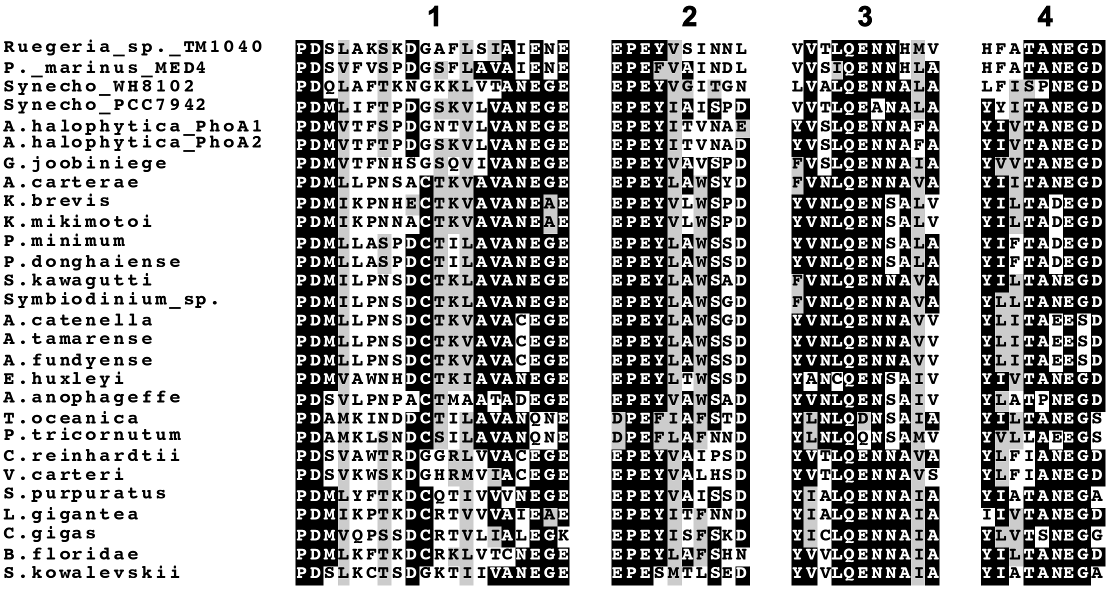

Supplementary Fig. 2. Alignment of four conserved domains of AP amino acid sequences shared by different marine organisms. Full species names and accession numbers of the sequences are shown in Fig.5. Identical residues are black-shaded while chemically similar ones gray-shaded.
